# Mitochondrial dynamics and antiviral responses in Atlantic salmon cardiomyocytes during piscine myocarditis virus infection

**DOI:** 10.1016/j.isci.2026.116831

**Published:** 2026-07-18

**Authors:** Antoni Malachowski, Deanna Lynn Wolfson, Randi Olsen, Alf Seljenes Dalum, Ida Sundvor Opstad, Krishna Agarwal, Roy Ambli Dalmo, Jaya Kumari Swain

**Affiliations:** 1 Norwegian College of Fishery Science, Faculty of Biosciences, Fisheries and Economics, UiT The Arctic University of Norway, Muninbakken 21, Tromsø 9037, Norway; 2 Department of Physics and Technology, UiT The Arctic University of Norway, Klokkargårdsbakken 35, Tromsø 9019, Norway; 3 Advanced Microscopy Core Facility, Department of Medical Biology, UIT The Arctic University of Norway, Sykehusveien 44, Tromsø 9019, Norway

**Keywords:** mitochondria, cardiomyopathy syndrome, CMS, piscine myocarditis virus, PMCV, cardiomyocytes, antiviral response, Atlantic salmon

## Abstract

Viral infections in higher vertebrates are known to remodel mitochondrial dynamics, which play a critical role in regulating immune responses, energy production and cellular homeostasis. Currently, understanding of mitochondrial dynamics during viral infection in teleost is limited. This study provides the first detailed investigation of mitochondrial responses to piscine myocarditis virus (PMCV) infection, the causative agent of cardiomyopathy syndrome (CMS) and a severe cardiac disease in Atlantic salmon causing economic losses in aquaculture. This study investigates the temporal effects of PMCV infection in cardiomyocytes on mitochondrial dynamics, associated molecular responses using fluorescence microscopy, transmission electron microscopy, histopathology and RT-qPCR. Distinct mitochondrial subpopulations with specific morphologies and spatial distributions were identified. PMCV infection induced marked mitochondrial remodeling, characterized by early fission, followed by swelling and elongation at peak viral RNA levels. These findings link viral kinetics and immune response to mitochondrial remodeling, providing mechanistic insight into CMS pathogenesis and cardiac health.

## Highlights

• PMCV infection remodels mitochondrial morphology and modulates gene expression
• Antiviral response and inflammation are closely linked to mitochondrial remodeling
• PMCV infection preferentially impacts intermyofibrillar mitochondrial morphology
• Atlantic salmon cardiomyocytes share architecture with other teleost species

## Graphical abstract

**Figure:**
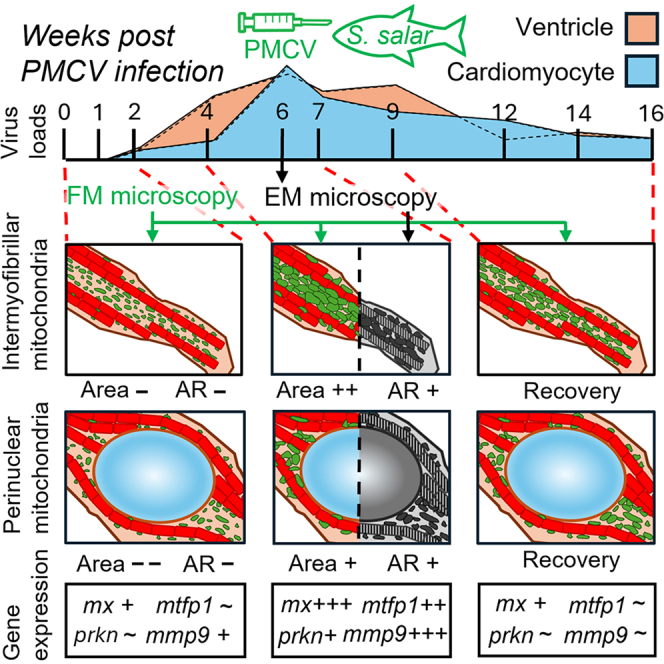

## Teaser

Cell biology; Ichthyology; Virology

## Introduction

Viral infections in mammalian host cells often target mitochondria, disrupting their dynamics and leading to dysfunction.^1^ Mitochondria are central to innate immune signaling, metabolism and the pathogenesis of viral infections. Many viruses manipulate mitochondrial fusion and fission to enhance replication, assembly and transmission while evading immune responses and inducing host cell damage. Restoring mitochondrial function has emerged as a potential therapeutic strategy for mitigating viral pathogenesis. While significant progress has been made in understanding mitochondrial dynamics during viral infections in mammals, the role of mitochondria in viral infections of teleosts remains poorly understood. This study provides the first detailed investigation of mitochondrial responses to viral infection in Atlantic salmon (*Salmo salar* L.), focusing on piscine myocarditis virus (PMCV) infection, the causative agent of cardiomyopathy syndrome (CMS). CMS is a severe cardiac disease in Atlantic salmon (*Salmo salar* L.) characterized by heart failure and high mortality (accounting to 8% of death caused by infectious diseases in Norwegian salmon aquaculture) leading to substantial economic losses.^2^ Not all PMCV infected fish develop CMS, as disease progression is likely influenced by factors such as host immune responses, genetics, stress and environmental factors.^2^ Thus, the mechanisms underlying progression of PMCV infection to CMS are poorly understood and unpredictable. CMS primarily affects larger, harvest-ready fish, with lower prevalence observed in newly sea-transferred smolts.^3^^,^^4^ PMCV can persist in the host asymptomatically, with a prolonged period between initial detection of viral RNA and the onset of clinical symptoms, during which fish may contain high viral RNA levels without external signs of disease. However, the timing of when PMCV infected fish become infectious remains unknown. Understanding the cellular and subcellular mechanisms of PMCV infection is critical not only for advancing knowledge of CMS pathogenesis but also for identifying potential biomarkers and therapeutic targets to mitigate its economic impact.

CMS mainly affects the spongious myocardium where studies have demonstrated that PMCV replication occurs primarily in cardiomyocytes, while endothelial cells in the heart do not show viral RNA.^5^ However, the direct effects of PMCV infection on cardiomyocyte structure and function, particularly at the organelle level (e.g., mitochondria), remain unexplored. This knowledge gap is partly due to lack of Atlantic salmon cardiomyocyte cell lines, which precludes the study PMCV infection *in vitro.*^5^^,^^6^ As an alternative, *ex vivo* cardiomyocytes isolated from digested heart tissue can be utilized as a biologically reliable stand-in.^7^ In this study, we utilized this approach to investigate the effects of PMCV infection on mitochondrial dynamics in Atlantic salmon cardiomyocytes.

Teleost cardiomyocytes exhibit a unique architecture distinct from their mammalian counterparts, reflecting adaptations to the physiological demands of aquatic environments. They tend to be smaller, slimmer, with tapered ends, and are typically mononuclear.^8^ Teleost mitochondria are less tightly packed between cardiac myofibrils and lack the hallmark periodicity observed in mammalian cardiac myocytes,^9^ and resemble the mitochondrial organization of mammalian neonatal cardiomyocytes.^10^ The myofibrils in teleost cardiomyocytes form a tubular structure that surrounds a central core of mitochondria, with the nucleus approximately positioned in the center,^8^ in contrast to a more uniform distribution of myofibrils and mitochondria in mammals.

Three distinct populations of mitochondria exist in mammalian cardiomyocytes-intermyofibrillar (IMF), perinuclear (PN), and subsarcolemmal (SS) mitochondria, each with a distinct role. While these subpopulations are well-characterized in mammals, their functional specialization and response to viral infections in teleost’s are still under-explored.^11^ IMF mitochondria constitute majority of the mitochondrial populations and exhibit higher calcium uptake and sensitive to oxidative stress.^12^ In contrast, PN mitochondria representing smaller population, exhibit higher mobility, fission/fusion events and are critical for IMF-mediated mitochondrial turnover.^12^^,^^13^ SS mitochondria primarily supply ATP for ion transport across the sarcolemma in mammals,^14^ but their role in teleosts is underexplored. SS mitochondria constitute a small percentage of total mitochondria in tuna cardiomyocytes, in contrast to red muscle myocytes.^15^

Mitochondrial dynamics is modulated by a complex mix of non-specific stressors, including reactive oxygen species and calcium overload, as well as specific molecular antiviral signaling.^15^^,^^16^ Disruption of calcium homeostasis can result in S100 protein dysregulation,^17^ as well as opening of the mitochondrial permeability transition pore, resulting in mitochondrial swelling.^18^ Activation of RIG-I and downstream MAVS can result in a drop in mitochondrial membrane potential,^19^ which can results in accumulation of PTEN-induced kinase 1 (PINK1) and parkin (PRKN) on the outer mitochondrial membrane.^20^ This activation initiates a ubiquitination cascade, leading to the selective clearance of damaged mitochondria through mitophagy, a process essential for preserving mitochondrial function and cardiac health. Mitophagy is conditionally preceded by fission of the mitochondrial network, which results in smaller mitochondria that can be more effectively engulfed by the autophagosome.^21^ Another important mediator of mitochondrial dynamics is mitochondrial fission protein 1 (MTFP1) which activates the fission ring and is crucial for initiation of mitophagy.^22^^,^^23^

Viral pathogens have evolved strategies to exploit mitochondrial dynamics to evade host immune responses. For instance, coxsackie virus B infection causes parkin (PRKN) translocation to mitochondria, which is blocked by knockdown of upstream mitochondrial sensor protein PINK1.^24^ Conversely, mitochondrial elongation, in contrast to fission, promotes a more effective anti-viral response. For example, Rig-I like receptor-mediated anti-viral signaling promotes mitochondrial elongation, which supports protective effects of mitochondrial antiviral protein MAVS.^25^^,^^26^ These findings highlight the dual role of mitochondrial dynamics in both viral evasion and host defense, underscoring the importance of studying these processes in the context of PMCV infection in Atlantic salmon.

In this study, we explored the dynamics of key mitochondrial and inflammatory genes to gain deeper insights into PMCV-induced cardiomyopathy. S100A1, a small calcium-sensing protein associated with mitochondrial F1-ATPase,^27^ has been identified as a critical regulator of cardiac contractile performance in mammals, particularly following cardiac infarction,^27^ suggesting it may be a useful biomarker for cardiac health following PMCV infection. Matrix metalloprotease 9 (MMP9) a well-known mediator of tissue remodeling during inflammation, is typically expressed in infiltrating immune cells in both higher vertebrates and teleost.^28^ In mammals, MMP-9 is overexpressed during the early stages of viral myocarditis and plays important role in disease progression.^29^ Similarly, in Atlantic salmon, MMP9 is highly upregulated during infection caused by various viruses, including salmonid alphavirus, infectious salmon anemia virus, and piscine reovirus.^30^^,^^31^ Previous microarray studies have reported matrix metalloprotease upregulation in the ventricle during PMCV infection,^32^^,^^33^ however, its precise role in PMCV pathogenesis and its potential as a biomarker for CMS progression remain poorly understood.

**Figure 1:**
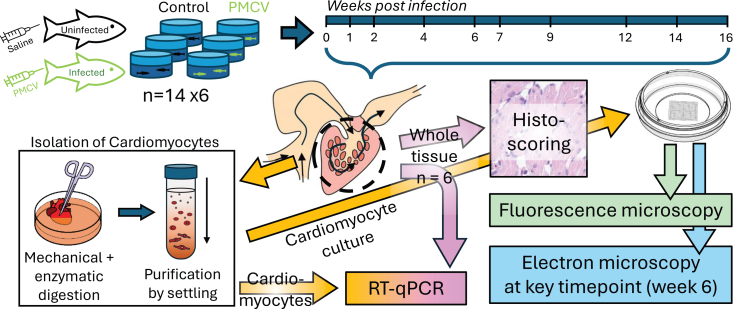
Experimental outline Atlantic salmon were infected via intramuscular injection, while control fish received saline injections. Fish were maintained in six tanks, with three tanks containing infected fish and three containing controls (14 fish per tank). Sampling was conducted at multiple time points between 1 and 16 weeks post-infection. At each time point, whole ventricle tissue was excised and divided into three portions: for RNA extraction, histopathology and for enzymatic digestion to isolate single-cell cardiomyocytes. Purified cardiomyocytes were obtained by low-speed centrifugation. A subset of isolated cardiomyocytes was used for fluorescence and electron microscopy imaging (n = 1–3), while the remaining portion was preserved for RNA isolation and further RT-qPCR analysis (n = 3–6). Ventricle tissue samples (*n* = 6) were also analyzed for histopathology. The microscopy dish image is provided courtesy of Ibidi GmbH.

**Figure 2:**
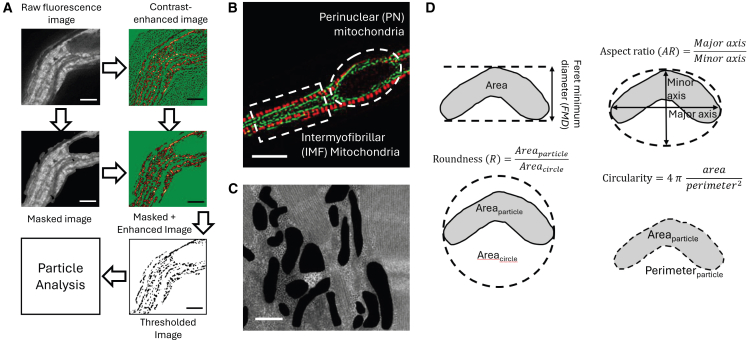
Microscopy and image analysis workflow (A) Image processing workflow: Live, *ex vivo* cardiomyocytes stained with MitoTracker Green (mitochondria) and CellMask Deep Red (actin) were prepared for volume imaging. Contrast was enhanced using a Difference-of-Gaussians transformation, and background was removed via variance-based masking. The processed output was thresholded using Otsu’s algorithm. To minimize cross-image correlation, analysis was subsampled to every third image. Scale bar: 3 μm. (B) Mitochondrial subtypes: Two mitochondrial subtypes were analyzed—intermyofibrillar (IMF) and perinuclear (PN) as demarcated by the dotted line. Scale bar: 8 μm. (C) Electron microscopy segmentation: Mitochondria in electron microscopy images were manually segmented to isolate individual particles for morphometric analysis. Scale bar: 500 nm. (D) Morphometric measurements: Five parameters were measured on segmented particles from fluorescence and electron microscopy images: area, Feret minimum diameter (*FMD*), roundness (*R*), aspect ratio (*AR*), and circularity. All processing and measurements were performed using ImageJ.

Our aim in this study was to bridge these knowledge gaps by investigating the effects of PMCV infection on mitochondrial dynamics, antiviral responses, and inflammation in Atlantic salmon ventricular cardiomyocytes. Using a modified mammalian heart digestion protocol, we isolated live cardiomyocytes and studied mitochondrial response to an *in vivo* infection until 16 weeks postinfection (WPI) using two microscopy modalities and RT-qPCR (Figure 1). Fluorescence microscopy was used for high-throughput morphological analysis of mitochondria, while in-depth morphology of the mitochondrial network was studied via transmission electron microscopy (Figure 2). Additionally, we evaluated degree of pathological changes using histology and analyzed viral RNA expression, antiviral gene expression, and markers of mitochondrial function and cardiac health in isolated cardiomyocytes and whole ventricular tissue using RT-qPCR. Our findings reveal dynamic and subtype specific changes in mitochondrial morphology and function during PMCV infection, with significant remodeling occurring prior to and during peak viral RNA levels. These changes were accompanied by correlation between antiviral responses, *mmp9* expression, viral kinetics and histopathology, providing important insights into the role of mitochondria in PMCV-induced CMS. This research establishes a foundation for future research into mitochondrial dynamics in Atlantic salmon cardiomyocytes.

## Results

### Viral RNA expression dynamics and associated pathology

**Figure 3:**
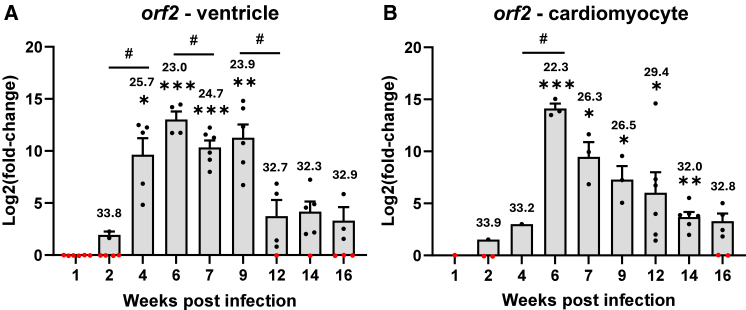
Viral RNA expression dynamics during PMCV infection (A) Virus RNA levels in whole ventricle tissue and (B) isolated ventricular cardiomyocytes were quantified using RT-qPCR, targeting ORF2 expression. Data are represented as mean ± SEM. *Y* axis represents virus RNA levels expressed as log2(fold-change) relative to 18S, following normalization with detection threshold (Ct = 36) using ΔΔCt Pfaffl method. Horizontal bars with hash symbol (#) indicate significant differences between time points (*p* < 0.05), while asterisks (∗*p* < 0.05, ∗∗*p* < 0.005, and ∗∗∗*p* < 0.0005) denote significant differences from the initial detection (2 WPI) using Mann-Whitney U test. Red dots along the *x* axis represent samples with undetectable viral RNA. Average Ct values are displayed above the bars.

Following experimental challenge of Atlantic salmon with PMCV via intramuscular injection, we observed a rise in whole ventricle tissue virus RNA levels, starting 2 WPI, with all tested ventricle tissue samples showing detectable viral RNA levels by 4 WPI (Figure 3A). Viral RNA levels in whole ventricle tissue plateaued between 4 and 9 WPI before declining from 12 WPI onwards, with progressive clearance of viral RNA levels. By 16 WPI, 50% of whole ventricle tissue samples (Figure 3A) and 33% of isolated cardiomyocyte samples (Figure 3B) no longer exhibited detectable viral RNA levels. No external clinical symptoms or mortality were observed throughout the study.

Viral RNA levels in isolated cardiomyocytes followed a similar temporal pattern to that observed in whole ventricle tissue. Viral RNA expression was first detected in isolated cardiomyocytes at 2 WPI, consistent with the timing observed in the ventricle (Figure 3B). At 4 WPI, viral RNA levels in isolated cardiomyocytes were lower than those in whole ventricle tissue. Peak viral RNA levels in cardiomyocytes were observed at 6 WPI, reaching levels comparable to those in whole ventricle tissue. However, the kinetics of viral expression diverged between whole ventricle tissue and isolated cardiomyocytes thereafter. While whole ventricular tissue viral RNA expression levels plateaued from 4 to 9 WPI, the virus RNA levels in isolated cardiomyocyte began to decline at 7 WPI. By 14 and 16 WPI, viral RNA levels in both isolated cardiomyocytes and the whole ventricle tissue samples converged to similar levels.

It is important to note that this study quantifies viral RNA expression levels in whole ventricle tissue and isolated cardiomyocytes but does not measure infectious virus particles or determine when infected fish become infectious.

**Figure 4:**
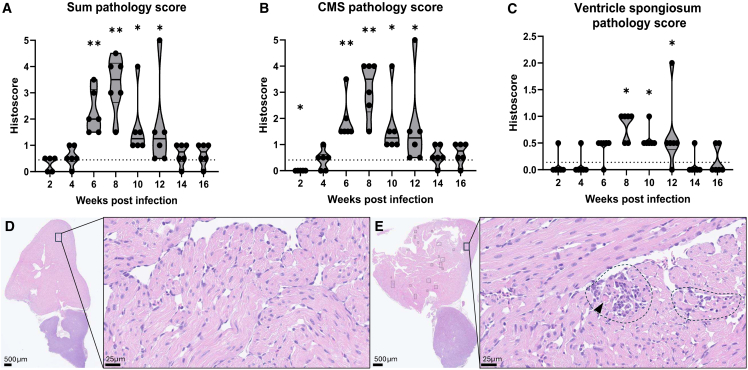
Histopathology scoring of Atlantic salmon heart following experimental infection with PMCV (A–C) Show histopathology scores throughout the course of the infection. Data are represented through violin plots displaying the median (shown as a central line) and the interquartile range. (A) Shows pathology score of the entire heart (Sum pathology score). (B) Shows CMS pathology scores of the heart relevant to PMCV, namely inflammation in the atrium and the ventricle-spongiosum (CMS pathology score). Similar scores at various time points in (A and B) indicate that majority of histopathological lesions are associated with CMS. (C) Pathology score confined to the ventricle spongiosum (ventricle spongiosum pathology score). The horizontal dotted lines in A–C represent the average histoscore in the uninfected group (A = 0.448, *n* = 48 samples, B = 0.413, *n* = 48 samples, C = 0.138, *n* = 47 samples). Significance between the infected and uninfected group was calculated at each time point (*n* = 6) using non-parametric Mann-Whitney test and significance level shown as asterisks (∗*p* < 0.05, ∗∗*p* < 0.005) above the violin plot. (D and E) Show representative hematoxylin-eosin (HE)- stained ventricle sections from uninfected fish (D) and piscine myocarditis virus (PMCV) infected fish 8 weeks post infection (E). (E) Shows moderate multifocal myocarditis in the ventricle as indicated by boxes. Insets in (D and E) show magnification of selected regions of the spongiosum showing normal tissue morphology in the uninfected fish (D) and multifocal mononuclear myocarditis (accentuated by dotted lines) of the spongious myocardium of the ventricle associated with necrosis of cardiomyocytes (arrowhead). Scale bars as indicated.

To investigate whether the progression of viral RNA levels contributed to CMS pathology, we performed histopathological scoring of PMCV infected hearts. The histopathological assessment reflected the viral RNA expression pattern although, as expected, at a delayed time frame, underscoring the fact that pathological lesions are preceded by the viremic phase. At 2 and 4 WPI no difference in pathology score was detected for the Sum pathology score (Figure 4A) compared to control fish. However, at 6 and 8 WPI (*p* < 0.005) and 10 and 12 WPI (*p* < 0.05), both the Sum pathology score and CMS pathology score (Figure 4B) were significantly higher in the infected group, while no significant differences were seen at 14 and 16 WPI (Figures 4A and 4B). When examining the spongious myocardium of the ventricle separately (target tissue of this study), a significantly higher ventricular spongiosum pathology score was found in the infected group at 8 and 10 WPI (Figure 4C). The CMS pathology score closely mirrored the Sum pathological score, indicating that majority of histopathological lesions were associated with CMS. Qualitatively, the observed lesions corresponded to those previously described for CMS,^34^ including mononuclear endocarditis and myocarditis in the spongious myocardium of the ventricle with increasing occurrence of degeneration and necrosis of cardiomyocytes (Figure 4E) and associated hypertrophy of the endocardium (Figure 4E) with increasing severity of inflammation. Only slight occurrence of mononuclear epicarditis in the ventricle was noted in some hearts. The overall contribution of spongious ventricle layer to CMS pathology was lower compared to the atrium which aligns with the typical progression of CMS, where the atrium is first affected. Specifically, the spongious ventricle pathology score remained low (score ∼1), corresponding to mild pathology (12.5%–25%) at 8 and 10 WPI (Figures 4C and 4E). No inflammation was noted in the spongious myocardium of the uninfected group (Figure 4D).

### Salmon cardiomyocyte cellular architecture

**Figure 5:**
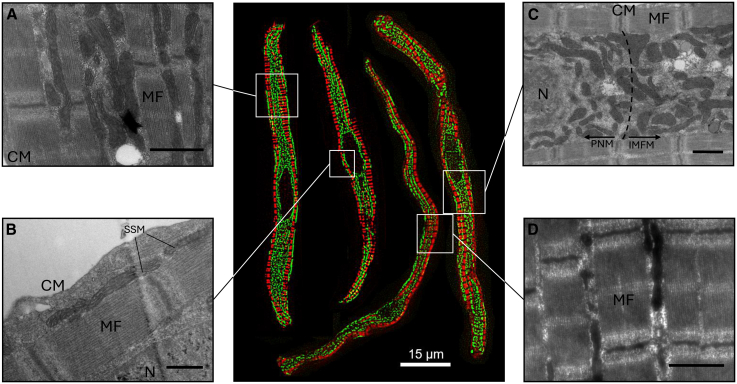
Structural organization of uninfected *ex vivo* ventricular cardiomyocytes at 6 weeks post-infection The central panel shows a composite fluorescence microscopy image of cardiomyocytes stained with MitoTracker Green (mitochondria) and CellMask Deep Red (actin). Insets correspond to regions highlighted in transmission electron micrographs (TEM) from other samples. Mitochondria are loosely packed in the cardiomyocyte core, surrounded by a myofibril sheath, and may align parallel to adjacent actin myofibrils. Fluorescence images were processed using difference-of-Gaussian (DoG) filtering and variance-based masking to enhance actin and mitochondrial channels, followed by merging the cleaned actin signal with the DoG-enhanced mitochondrial channel. Four locations were selected for emphasis. (A) Mitochondria arranged between myofibrils (MF), oriented roughly parallel to the myofibrils. A circular void at the bottom likely represents a lipid droplet dislodged during ultra-fine sectioning. (B) A small fraction of mitochondria are positioned directly beneath the cell membrane (CM), labeled as subsarcolemmal mitochondria (SSM), with a visible nucleus (N). (C) Loosely packed mitochondria within the cardiomyocyte core, surrounded by a myofibril sheath. Mitochondria near the nucleus, identified as perinuclear mitochondria (PNM), while those further away are classified as intermyofibrillar mitochondria (IMFM), both indicated by arrows. The dotted line demarcates an arbitrary boundary between the PNM and IMFM regions. (D) A fraction of mitochondria within the myofibril sheath showing mammalian-like orientation, with tight contact between mitochondria and myofibrils. All TEM images were acquired from resin-embedded, stained 70 nm sections (see STAR Methods for details). Scale bars: 1000 nm (A–C) and 500 nm (D).

**Figure 6:**
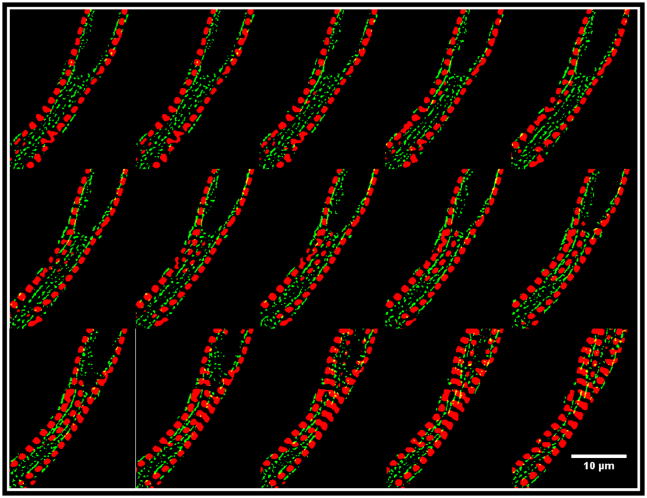
Representative fluorescence microscopy image stack of an Atlantic salmon cardiomyocyte Fifteen consecutive slices from a volume image are shown, starting at the top left and ascending from left to right. Cardiomyocytes were isolated from ventricle tissue via enzymatic digestion, cultured, and stained with MitoTracker prior to imaging. Slice-by-slice images and 3D projections of entire cells are shown in Videos S1, S2, S3, and S4. Image processing included difference-of-Gaussian (DoG) filtering, variance-based masking, and Otsu thresholding to enhance actin and mitochondrial channels while suppressing background. The cleaned actin signal was merged with the DoG-enhanced mitochondrial channel for visualization. Scale bar: 10 μm.

Fluorescence microscopy of isolated cardiomyocytes revealed characteristic structural features and subcellular organization patterns. The cells exhibited a predominantly spindle morphology, with widths slightly exceeding nuclear dimensions (110%–140% of nucleus width), displaying occasional bulging in the perinuclear (PN) region (Figures 5 and 6, Videos S1, S2, S3, and S4). The cardiomyocytes measured approximately 8 μm in width, ranging from 6 to 12 μm, and had an average length of 100 μm, with a range of 50–150 μm. Nuclear positioning was variable, with most nuclei located toward the geometric center of the cell, although some were observed closer to cell termini.

Video S1. Volume image stack of ex-vivo uninfected cardiomyocytes, related to Figure 6 Green shows mitochondria (MitoTracker Green) and red shows actin (CellMask Deep Red). Images were processed using Difference-of-Gaussian (DoG) filtering, variance-based masking, and adaptive thresholding to suppress background and enhance viewability. Corresponding 3D projections are provided in Video S3.

Video S2. Volume image stack of ex-vivo infected cardiomyocytes, related to Figure 6 Green shows mitochondria (MitoTracker Green) and red shows actin (CellMask Deep Red). Images were processed using Difference-of-Gaussian (DoG) filtering, variance-based masking, and adaptive thresholding to suppress background and enhance viewability. Corresponding 3D projections are provided in Video S4.

Video S3. 3D projections of uninfected ex-vivo cardiomyocytes, related to Figure 6 Mitochondria were stained with MitoTracker Green. Images were processed using rolling ball background subtraction, despeckle, and 3D DoG filtering to suppress background and enhance viewability. Depth-colorization was applied in 12-slice color blocks (yellow → orange → red → magenta), and a brightest-point 3D projection was generated. Corresponding image stacks are provided in Video S1.

Video S4. 3D projections of infected ex-vivo cardiomyocytes, related to Figure 6 Mitochondria were stained with MitoTracker Green. Images were processed using rolling ball background subtraction, despeckle, and 3D DoG filtering to suppress background and enhance viewability. Depth-colorization was applied in 12-slice color blocks (yellow → orange → red → magenta), and a brightest-point 3D projection was generated. Corresponding image stacks are provided in Video S2.

The subcellular organization of cytoskeletal and organellar components displayed a characteristic pattern. Actin fibers formed a peripheral sheath around the cellular core, which was densely populated with mitochondria. In some cells, singular myofibrils were observed crossing the mitochondria-rich core, though this occurred infrequently. Occasionally, myofibrils were observed twisted along the longitudinal axis of the cell or forming curled cell termini with irregular structural patterns. FM results showed that cardiomyocytes on average contained approximately two thousand (2070.95 ± 465.7, *n* = 119) mitochondria.

When comparing the cardiomyocyte architecture between control and infected groups, infected group cardiomyocytes seemed to have slightly increased cell width and round ends (Figure 6 and Videos S1, S2, S3, and S4). These alterations might suggest structural remodeling in response to infection.

### Morphological differences of two subtypes of cardiac mitochondria

Microscopic examination of *ex vivo* Atlantic salmon cardiomyocytes revealed three distinct mitochondrial subpopulations based on their subcellular localization: perinuclear, intermyofibrillar (IMF), and subsarcolemmal (SS) mitochondria (Figure 5).

The IMF mitochondria exhibited consistent parallel orientation relative to the myofibrils. TEM analysis of 2D images revealed IMF mitochondria were loosely packed, with gaps between myofibrils often filled with cytoplasm and glycogen granules (Figure 5). Morphometric analysis of control group showed that IMF mitochondria were significantly larger than PN mitochondria, with an area approximately 8% greater and a Feret minimum diameter *(FMD)* 16% larger. The *AR* of IMF mitochondria was 11% higher than that of PN mitochondria, which together with 7% smaller circularity indicates a more elongated and irregular morphology of IMF mitochondria (Figure S4). In addition, PN mitochondria were typically arranged parallel to the nuclear perimeter, with perpendicular orientation observed only rarely (Figure 5C). Highly circular mitochondria were more frequent in this region. FM data showed that about 26.7% ± 4.7% mitochondria constitute PN mitochondria per cell (*n* = 211).

In addition to PN and IMF mitochondria, a small subpopulation of SS mitochondria was identified beneath the sarcolemma using both electron and fluorescence microscopy. These mitochondria were positioned between the myofibrils and the cell membrane and exhibited a characteristic narrow and elongated morphology, with their long axes oriented parallel to the sarcolemma. No trend in relative localization was found. Due to the small population size, SS mitochondria were not analyzed separately in this study.

### Mitochondria from PMCV infected group exhibit morphological changes compared to uninfected

We used 2D FM and TEM images to investigate the effect of PMCV on mitochondrial morphology within cardiomyocytes. Segmented mitochondria were classified according to their spatial location within the cardiomyocyte as shown in Figure 2D. In this study, individual mitochondrial morphometric evaluation was done for whole cell, which was further grouped into IMF mitochondria and PN mitochondria.

**Figure 7:**
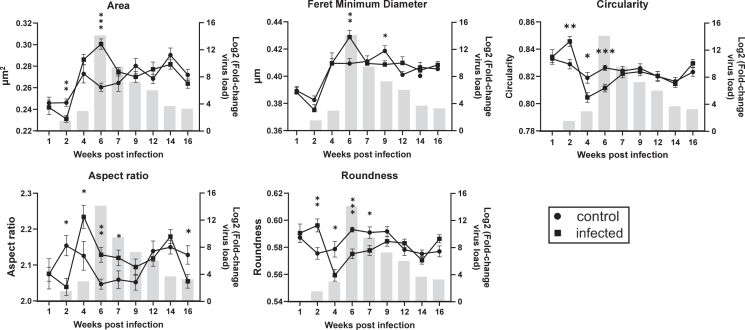
Morphometric analysis of mitochondria in *ex vivo* ventricular cardiomyocytes during PMCV infection Mitochondrial morphometrics were analyzed in cardiomyocytes isolated from Atlantic salmon ventricle tissue at multiple time points post PMCV infection. Cardiomyocytes were purified, cultured, and stained with MitoTracker Green prior to imaging for fluorescence microscopy, and entire cells were imaged and analyzed using ImageJ. Mitochondrial sizes were calculated per cell. Data are represented as mean ± SEM and are shown as points on the line graph (left *y* axis). Virus RNA levels, measured via RT-qPCR, are displayed as bars in the background (right *y* axis). Statistical significance is indicated by ∗*p* < 0.05, ∗∗*p* < 0.005, and ∗∗∗*p* < 0.0005, determined using the Mann-Whitney U test. A total of 234 cells were analyzed in the infected group and 233 cells in the uninfected group, with 1–3 fish sampled per group per time point. Results from PN and IMF mitochondria subsets can be found in Figures S1A and S1B, respectively.

Quantitative analysis of 2D mitochondrial networks in *ex vivo* ventricular myocytes revealed significant infection-induced alterations in mitochondrial morphology during the early and peak phases of PMCV infection (2–7 WPI) (Figure 7). At 2 WPI, mitochondria in cardiomyocytes of infected fish were significantly smaller (6% reduction in area) and exhibited a rounder morphology (5% lower *AR*, and 4% higher *R* and 2% higher circularity) compared to the control group. By 6 WPI, this trend reversed, with mitochondria in the infected group showing a marked increase in size (15% larger area) and width (5% greater *FMD*), as well as becoming more elongated (4% higher *AR*, and 3% lower *R* and 2% lower circularity) relative to the uninfected group. This pattern of mitochondrial swelling, elongation and irregular morphology persisted from 4 to 7 WPI, with the most pronounced differences observed at 6 WPI. These trends were consistent across both PN and IMF mitochondrial sub-types (Figure S1). Differences in mitochondrial morphology become insignificant following the 9 WPI time point.

**Figure 8:**
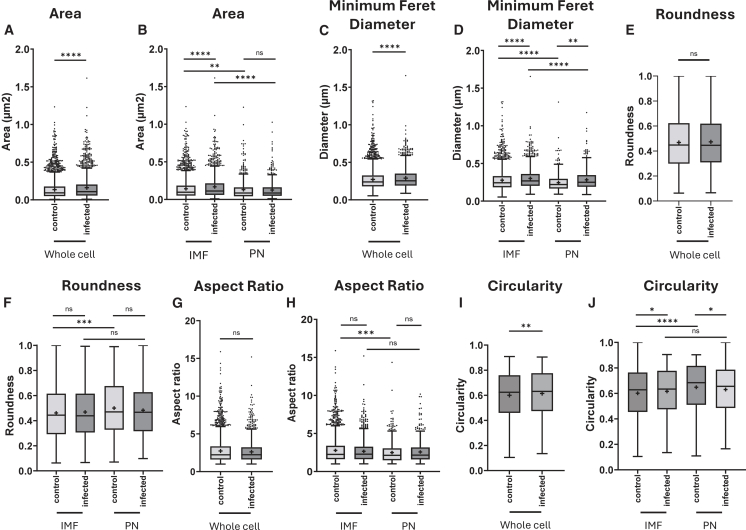
Morphometric analysis of mitochondria from *ex vivo* ventricular cardiomyocytes using electron microscopy Cardiomyocytes were isolated from Atlantic salmon ventricles, purified, cultured, fixed, and embedded for electron microscopy. Mitochondria were analyzed across entire cell (A, C, E, G, and I) and further classified into intermyofibrillar (IMF) or perinuclear (PN) subpopulations based on their distance from the nucleus (B, D, F, H, and J). Mitochondria within 2 μm of the nucleus were classified as PN, while those beyond this threshold were classified as IMF. Each data point represents an individual mitochondrion. Statistical significance is indicated as ns = not significant, *p* > 0.05, ∗∗*p* < 0.005, ∗∗∗*p* < 0.0005, and ∗∗∗∗*p* < 0.00005, calculated using the Mann-Whitney U test. Boxplots are represented by median, interquartile range (IQR), whiskers extending to data within 1.5×IQR, and individual outliers represented as points. The group means are indicated by a plus symbol (+). A comparison of PN and IMF mitochondria in control group can be found in Figure S2.

Morphometric evaluation of TEM images at 6 WPI, the time point corresponding to peak viral expression and the most pronounced infection-induced changes observed in FM and transcript data, revealed significant alterations in mitochondrial morphology between the control and infected groups (Figure 8). The total mitochondria population in the infected group exhibited an 18% increase in area, suggesting mitochondrial swelling or fusion state as a response to infection. Additionally, mitochondria from infected fish displayed 6% increase in *FMD*, consistent with FM findings, and 3% increase in circularity. However, despite these changes in size, no significant differences in mitochondrial shape parameters, such as *AR* (4% decrease) and *R*, were detected in the total mitochondrial population between infected and control groups at 6 WPI.

Further analysis of mitochondrial subpopulations revealed distinct subtype-specific responses to PMCV infection. At 6 WPI, IMF mitochondria in the infected group exhibited a swollen morphology, characterized by a significant 18% increase in area, 6% increase in *FMD*, 2% increase in circularity, and a 4% decrease in *AR* compared to their respective controls (Figure 8), suggesting more fragmented and fission state. In addition, the ratio of PN to IMF populations increased by three times in the infected compared to uninfected group. These changes suggest that IMF mitochondria are more sensitive to PMCV-induced stress. In contrast, PN mitochondria appeared to be also affected, but to a lesser degree, displaying a marginal 4% decrease in both area and *R*, 2% increase in circularity alongside a 10% increase in *FMD* and a 3% increase in *AR*, indicating slight elongation and increased dimensions. The infection also abrogated differences in *AR*, *R*, and circularity between PN and IMF mitochondria, indicating a shift in PN mitochondrial dynamics toward a more active state.

Under uninfected conditions, IMF mitochondria were significantly larger and exhibited higher *AR* and lower *R* and circularity compared to PN mitochondria, reflecting their characteristic elongated morphology (Figure S2). However, these distinct differences between mitochondrial sub-types were completely abrogated in the infected group (Figure 8). The infection-induced swelling and alterations in size and shape of both PN and IMF mitochondria led to a convergence in their morphometric characteristics, suggesting a compensatory response to mitigate cellular stress and maintain mitochondrial function during PMCV infection.

### Gene expression

**Figure 9:**
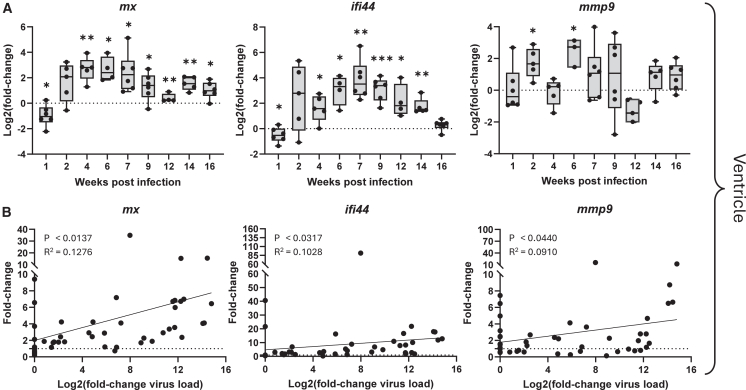
Expression and correlation analysis of antiviral and inflammatory markers in whole ventricle tissue (A) Gene expression levels of antiviral and inflammatory markers in whole ventricle tissue were quantified using RT-qPCR (*n* = 6) and analyzed using the ΔΔCt Pfaffl method. Data are presented as fold-change relative to controls. Statistical significance is indicated as ∗*p* < 0.05, ∗∗*p* < 0.005, and ∗∗∗*p* < 0.0005, calculated using parametric *t* test or Mann-Whitney U test wherever applicable. Boxplots shows individual data points, with the box representing the interquartile range (IQR), the median shown as a horizontal line, and whiskers extending to the minimum and maximum values. Data are represented as the median with whiskers. (B) Correlation analysis between gene expression and viral RNA expression (log2 fold-change) was performed using linear regression on data pooled across all time points. The significance of the linear fit was tested against the null hypothesis, with the *p* value and R^2^ value provided in the inset. Spearman’s rank correlation analysis across all gene expression data can be found in Figure S3A.

**Figure 10:**
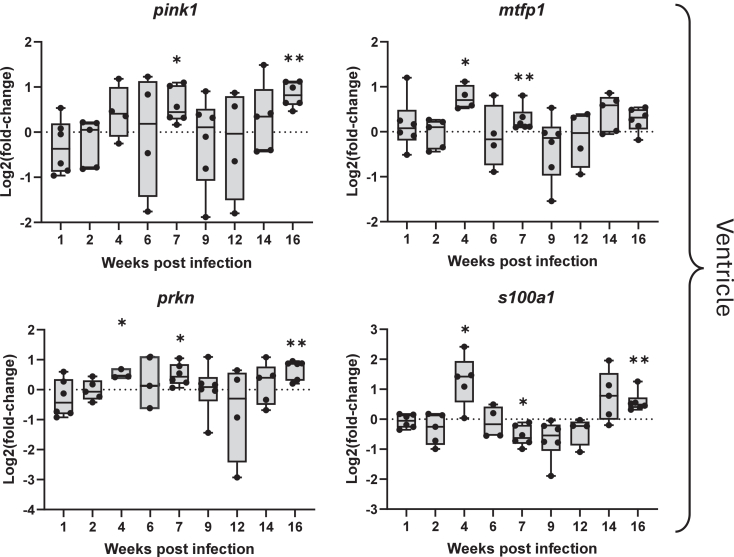
Expression of mitochondrial markers in whole ventricle tissue Gene expression of mitochondrial markers in whole ventricle tissue was quantified using RT-qPCR (*n* = 6) and analyzed using the ΔΔCt Pfaffl method. Results are presented as fold-change relative to controls. Statistical significance is indicated as ∗*p* < 0.05 and ∗∗*p* < 0.005. Boxplots show individual data points, with the box representing the interquartile range (IQR), the median shown as a horizontal line, and whiskers extending to the minimum and maximum values. Data are represented as the median with whiskers. Spearman’s rank correlation analysis across all gene expression data can be found in Figures S3A and S4.

**Figure 11:**
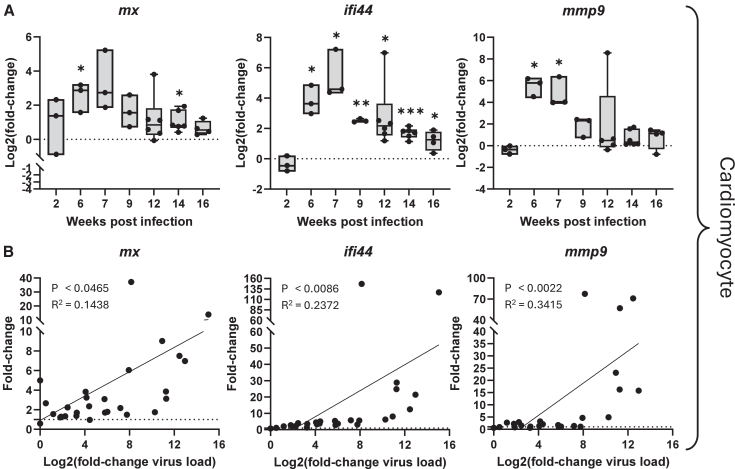
Expression and correlation analysis of antiviral and inflammatory markers in isolated cardiomyocytes (A) Gene expression levels of antiviral and inflammatory markers in isolated cardiomyocytes were quantified using RT-qPCR (*n* = 3–6) and analyzed using the ΔΔCt Pfaffl method. Data are presented as fold-change relative to controls. Statistical significance is indicated as ∗*p* < 0.05, ∗∗*p* < 0.005, and ∗∗∗*p* < 0.0005. Boxplots show individual data points, with the box representing the interquartile range (IQR), the median shown as a horizontal line, and whiskers extending to the minimum and maximum values. Data are represented as the median with whiskers. (B) Correlation analysis between gene expression and viral RNA expression (log2 fold-change relative to 18S) was performed using linear regression on data pooled across all time points. The significance of the linear fit was tested against the null hypothesis, with the *p* value and R^2^ value provided in the inset. Spearman’s rank correlation analysis across all gene expression data can be found in Figure S3B.

**Figure 12:**
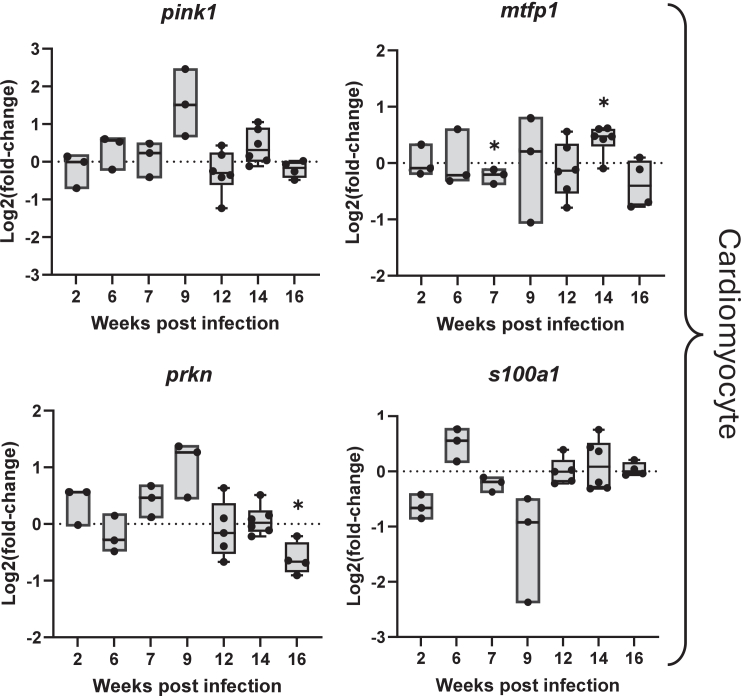
Expression of mitochondrial markers in isolated cardiomyocytes Expression levels of mitochondrial markers in isolated cardiomyocytes were quantified using RT-qPCR (*n* = 3–6) and analyzed using the ΔΔCt Pfaffl method. Results are presented as fold-change relative to controls. Statistical significance is indicated as ∗*p* < 0.05. Boxplots show individual data points, with the box representing the interquartile range (IQR), the median shown as a horizontal line, and whiskers extending to the minimum and maximum values. Data are represented as the median with whiskers. Spearman’s rank correlation analysis across all gene expression data can be found in Figures S3B and S4.

To further study the effect of PMCV infection on antiviral, inflammatory response and mitochondrial function at the transcript level, RT-qPCR was performed on whole ventricle tissue (Figures 9 and 10) and isolated cardiomyocytes (Figures 11 and 12) obtained from the same fish. The analysis revealed distinct trends in gene expression associated with PMCV infection, particularly across antiviral genes, genes related to mitochondrial function (based on mammalian studies) and inflammatory markers. Antiviral response genes *mx* and *ifi44* exhibited sustained and significant upregulation in both ventricular tissue and isolated cardiomyocytes. In whole ventricle tissue, these genes were upregulated from 2 to 16 WPI (Figure 9A), while in isolated cardiomyocyte, upregulation was observed from 6 to 16 WPI, peaking at 6–7 WPI (Figure 11A). This suggests a prolonged antiviral state, which may contribute to the resolution of infection. Notably, the expression of *mx*, *ifi44*, and *mmp9* was significantly correlated with viral RNA levels in both whole ventricle tissue (Figure 9B) and isolated cardiomyocytes (Figure 11B).

Mitochondrial function related genes, including *pink1*, *mtfp1*, and *prkn*, displayed a similar biphasic pattern of upregulation in whole ventricle tissue from 4 to 7 WPI (Figure 10), except during early stages of infection (1 and 2 WPI), showing their close mitochondrial functional relationship. Absence of upregulation of mitophagy-related genes (Figures 10 and 12) during early stages of infection (1–2 WPI), combined with reduced mitochondrial size (Figure 7) suggests that clearance of damaged mitochondria may be impaired or delayed. Spearman’s rank correlation analysis showed significant correlations between the expression of these mitochondrial genes in the ventricle (r = 0.74–0.82) across all time points (Figure S3A). In isolated cardiomyocytes, the expression of *mtfp1*, *pink1*, and *prkn* was also significantly correlated (Figure S3B). However, the expression of *pink1*, *mtfp1*, and *prkn* were not significantly correlated to viral RNA levels (Figure S4) but were significantly correlated to antiviral response via *mx* gene.

The inflammatory marker *mmp9* exhibited prolonged upregulation in whole ventricle tissue, as early as 2 WPI, persisting through 16 WPI (Figure 9). Two exceptions were observed at 4 and 12 WPI, where *mmp9* upregulation in whole ventricle tissue returned to baseline or was downregulated. In isolated cardiomyocytes, *mmp9* expression was significantly upregulated, peaking at 6 WPI with a 45-fold increase, followed by a decreasing trend from 9 WPI onwards (Figure 11A). Overall, *mmp9* expression was more pronounced in isolated cardiomyocytes compared to whole ventricle tissue and had a stronger correlation with viral RNA levels (Figures 9 and 11).

The calcium-binding protein *s100a1* displayed a distinct triphasic expression profile in both whole ventricle tissue and isolated cardiomyocyte during PMCV infection (Figures 10 and 12). *s100a1* expression was initially downregulated until 2 WPI and was significantly upregulated at 4 and 16 WPI, whereas a marked downregulation was observed between 7 and 12 WPI compared to the control group (Figure 10). In isolated cardiomyocytes, *s100a1* expression showed initial upregulation at 6 WPI, followed by a downregulation at 7–9 WPI, before returning to baseline levels from 12 WPI onwards (Figure 12).

## Discussion

Our study is the first to characterize and track mitochondrial dynamics of distinct mitochondrial subpopulations in Atlantic salmon cardiomyocytes during viral infection, with implications for understanding the pathogenesis of cardiomyopathy syndrome (CMS). By employing advanced imaging techniques and molecular analyses, we demonstrate that PMCV infection induces remodeling of mitochondrial morphology and function, which aligns with viral RNA expression, mild CMS pathology, antiviral response, and inflammation. These findings contribute to the growing body of knowledge on the role of mitochondria in viral infections and highlight infection biology during CMS progression in Atlantic salmon.

### Structural organization of Atlantic salmon cardiomyocytes

Fluorescence imaging of whole Atlantic salmon cardiomyocytes revealed narrow cells with tapered termini and a myofibril sheath surrounding a core of mitochondria. This structural organization is consistent with findings from other teleost species, including Pacific bluefin tuna, European plaice, and rainbow trout^8^^,^^35^^,^^36^ (Figure 5 and Videos S1, S2, S3, and S4). However, studies in rainbow trout have reported a smaller subpopulation (10%) of wider, sheet-like cardiomyocytes,^37^ which were not observed in the current study. The narrower size of teleost cardiomyocytes may represent an evolutionary adaptation to facilitate the formation of very thin ventricular trabeculae, which enhance oxygen uptake from surrounding blood by diffusion.^38^

Electron microscopy further revealed that mitochondria in *ex vivo* Atlantic salmon cardiomyocytes are concentrated along the center line of the cardiomyocyte, surrounded by a sheath of myofibrils, with true intermyofibrillar (IMF) mitochondria being relatively uncommon (Figure 5). This finding aligns with results reported in rainbow trout^9^^,^^35^ and neonatal mammals.^10^ Even when teleost IMF mitochondria, including Atlantic salmon, which constitute the major subpopulation, where arranged parallel to the myofibrils—they did not display the lattice-like arrangement of mitochondria as observed in mammalian cardiomyocytes.^39^ Our results on mitochondrial morphology near the nucleus, representing PN mitochondria, are consistent with those reported in the rat heart, indicating that PN mitochondria are smaller, rounder, and less numerous than IMF mitochondria (Figure 8).^40^ These structural differences between teleost and mammalian cardiomyocytes likely reflect adaptations to the unique physiological demands of aquatic environments, such as lower oxygen availability and higher reliance on diffusion for oxygen delivery. Furthermore, the differential responses of Atlantic salmon IMF and PN mitochondria to PMCV infection in this study are comparable to those observed in mammalian cardiomyocytes,^13^ highlighting their functional specialization.^41^

In addition to IMF and PN mitochondria, we also observed mitochondria positioned directly beneath the cell membrane (Figure 5), resembling mammalian subsarcolemmal mitochondria (SSM).^40^ However, these mitochondria comprised only a small fraction of the total mitochondrial population in Atlantic salmon cardiomyocytes. Subsarcolemmal mitochondria in teleost red muscle have been reported previously by Luther et al., who observed their presence via electron microscopy in *Cyprinus carpio*.^11^ Similarly, another study reported that while tuna red muscle has a high proportion of SSM, only 7% of mitochondria in the cardiac muscle are subsarcolemmal.^42^ Our imaging dataset corroborates these findings, showing that subsarcolemmal mitochondria are infrequent in Atlantic salmon cardiomyocytes and that their distribution appears random relative to the nucleus. The limited presence of SSM in teleost cardiomyocytes may reflect differences in energy demands and ion transport requirements compared to mammalian cardiomyocytes, where SSM play a critical role in maintaining ion homeostasis.^40^

### Mitochondrial remodeling during PMCV infection

Our findings reveal distinct temporal changes in mitochondrial morphology and function during PMCV infection, highlighting the dynamic interplay between viral replication, inflammation and host mitochondrial responses. Early in the infection (2 WPI), mitochondria exhibited reduced size and increased roundness, indicative of mitochondrial fission.^43^ This aligns with studies in mammalian systems, where viral infections exploit mitochondrial fission to evade host immune responses.^44^^,^^45^

The altered mitochondrial morphology observed during the early stages of infection (1–2 WPI), combined with the absence of upregulation of mitophagy-related genes (*pink1* and *prkn*), suggests that PMCV infection disrupts mitochondrial dynamics. The lack of mitophagy induction may impair mitochondrial quality control, leading to the accumulation of damaged mitochondria and exacerbating cellular stress. This disruption in mitochondrial homeostasis could represent a viral strategy to subvert host defenses, as has been reported in other viral infections where stalled mitophagy enhances viral replication and immune evasion.^46^^,^^47^ At peak viral RNA levels (6 WPI), we observed mitochondrial swelling, elongation, and irregular morphology, particularly in intermyofibrillar (IMF) mitochondria. These changes suggest a shift in mitochondrial dynamics toward mitochondrial fusion, potentially as a compensatory response to infection-induced stress and to support increased energy demands and cellular repair processes. Similar responses have been described in other systems, such as human respiratory syncytial virus (RSV), where mitochondrial elongation serves as a compensatory response to viral stress, aimed at maintaining energy production and reducing mitochondrial damage.^48^ Furthermore, mitochondrial elongation has also been associated with enhanced antiviral signaling, particularly through the activation of mitochondrial antiviral signaling protein (MAVS), which plays a critical role in innate immune responses.^25^

The observed convergence of morphometric characteristics between perinuclear (PN) and IMF mitochondria during peak infection further highlights the disruption of mitochondrial specialization. This convergence, combined with the mild CMS pathology observed in the spongious layer of the ventricle, suggests that PMCV infection impairs the cardiomyocytes’ ability to meet energy demands and maintain mitochondrial homeostasis, contributing to CMS progression. The mild degree of inflammation and CMS lesions observed in the spongious layer of the ventricle (12.5%–25% affected myocardium) further reflect the subclinical nature of the experimental model, providing insights into early mitochondrial responses to PMCV infection that may precede clinical disease.

These findings suggest that PMCV infection induces a biphasic mitochondrial response, with early fission potentially facilitating viral replication and immune evasion, followed by fusion as a host-driven compensatory mechanism to mitigate cellular damage, pathology and restore mitochondrial function. The interplay between mitochondrial fission, fusion, and antiviral signaling underscores the significant role of mitochondrial dynamics in the pathogenesis of CMS. Further studies are needed to explore the molecular pathways regulating these processes and their potential as therapeutic targets for mitigating CMS progression.

### Antiviral response and mitochondrial function

The upregulation of antiviral genes (*mx* and *ifi44)* in both whole ventricle tissue and isolated cardiomyocytes underscores the activation of innate immune responses during PMCV infection. The sustained expression of these genes, even when viral RNA levels decline, suggests a prolonged antiviral state, which may contribute to the resolution of infection. However, it is important to note that gene expression does not always directly correlate with the expression of functional proteins involved in physiological responses. The correlation between antiviral gene expression and mitochondrial dynamics highlights the interplay between mitochondrial function and immune responses. Notably, the upregulation of *mtfp1*, *pink1*, and *prkn* in whole ventricle tissue, and *pink1* and *prkn* in isolated cardiomyocytes during peak infection suggests that mitochondrial fission and mitophagy may play a role in regulating the antiviral response, as observed in mammalian studies.^21^^,^^22^ However, the absence of mitophagy related gene (*pink1* and *prkn*) upregulation during early infection, despite altered mitochondrial morphology, indicates a disruption in mitochondrial quality control. Such disruption of mitophagy has been reported as a viral strategy to evade host defenses in other systems^45^^,^^47^^,^^48^ Although the correlation between mitochondrial markers supports the observed temporal shifts, additional studies are required to validate these findings and further investigate the molecular mechanisms underlying these changes.

Mitochondrial dysfunction during early PMCV infection may also have broader implications for immune regulation. Mitochondria are critical hubs for certain innate immune signaling pathways, and their dysfunction may impair the production of antiviral cytokines, such as interferons, which are essential for controlling viral replication.^24^^,^^48^^,^^49^ This aligns with our previous study on whole ventricle tissue, where expression of interferon alpha (*ifna*) was downregulated until 4 WPI and T cell mediated immune response was suppressed until 2 WPI during PMCV infection.^49^ Mitochondrial dysfunction may explain the delayed upregulation of *ifi44* in isolated cardiomyocytes compared to whole ventricle tissue, as an initial suppression of mitophagy could hinder the activation of mitochondrial antiviral pathways.^50^^,^^51^

The inflammatory marker *mmp9* exhibited modest upregulation in isolated cardiomyocytes, peaking at 6 WPI which aligned with the peak viral RNA and antiviral gene expression. This finding is consistent with previous studies in teleost and mammals, where *mmp9* is implicated in tissue remodeling and inflammation during viral myocarditis.^28^^,^^29^ The correlation of *mmp9* expression with viral RNA levels and antiviral response followed by increased pathology at 8 WPI, suggests its potential as a biomarker for CMS progression. However, transient downregulation of *mmp9* at certain time points in ventricle tissue may reflect the mobility of different cell types at the site of virus infection. Further investigation is needed to elucidate its role in resolving inflammation. Additionally, correlation of *mmp9* with *mtfp1* expression (Figure S2B) highlights its association with mitochondria function. This is consistent with recent studies showing that *mmp9* can become closely associated with intracellular organelles including mitochondria.^52^ The clear upregulation of *mmp9* in cardiomyocytes from PMCV infected fish suggests that it may contribute directly to heart dysfunction, through mitochondrial damage and inflammation.

The triphasic expression profile of *s100a1*, a calcium-binding protein associated with mitochondrial ATP production, provides additional insights into the impact of PMCV infection on cardiac health. The initial downregulation of *s100a1* during early infection may reflect impaired mitochondrial function, while its upregulation at later stages suggests a compensatory mechanism to restore energy production. Similar patterns have been observed in mammalian models of cardiac injury, where *s100a1* levels correlate with contractile performance.^53^ These findings highlight the potential role of *s100a1* as a biomarker for cardiac health in PMCV infected salmon.

Although the fold changes observed for some genes, such as *pink1*, *mtfp1*, *prkn*, and *s100a1* are modest, they correlate well with the mild pathology observed in the ventricle. Even small changes in gene expression can have significant biological implications, reflecting early adjustments in mitochondrial fission and mitophagy that are critical for maintaining cellular homeostasis during infection.

In summary, our findings underscore the importance of mitochondrial dynamics in viral pathogenesis and demonstrate that PMCV infection induces dynamic changes in mitochondrial morphology and function in Atlantic salmon cardiomyocytes, which correlate with antiviral and inflammatory responses. The changes in mitochondrial subpopulation dynamics and the prolonged upregulation of inflammatory markers highlight the complex interplay between mitochondrial dynamics and immune responses during viral infections. These insights provide a foundation for future research into the pathogenesis of CMS and other viruses, and the development of molecular diagnostics to combat this economically significant disease.

### Limitations of the study

While this study provides valuable insights into mitochondrial dynamics and antiviral responses in Atlantic salmon cardiomyocytes during PMCV infection, several limitations should be acknowledged. Future studies incorporating RNA-seq could provide a more comprehensive understanding of transcriptional changes associated with PMCV infection. The lack of clear cristae resolution in electron microscopy prevented analysis of mitochondrial cristae structure, limiting our ability to assess a critical aspect of mitochondrial health. Additionally, the small size of fish at early time points restricted the quantity of isolated cardiomyocytes and insufficient RNA yield, preventing RT-qPCR analysis of selected genes at 1 and 4 WPI and leaving an incomplete picture of early gene modulation. Functional assays to measure mitochondrial bioenergetics, such as ATP production or reactive oxygen species generation, were not performed due to sample limitations, which would have provided a more comprehensive understanding of mitochondrial function. Finally, extending the study beyond 16 WPI could reveal whether a long-term PMCV infection results in viral clearance or resurgence. Most importantly, we acknowledge that the absence of a PMCV-negative tissue homogenate control is a limitation of this study. While such a control would isolate possible specific effects of PMCV, the observed mitochondrial and inflammatory responses in the current study cannot be attributed exclusively to PMCV. Instead, these responses should be interpreted as being induced by a PMCV-containing cardiac tissue homogenate, where PMCV is likely the primary immunostimulatory factor but not the sole potential contributor. However, previous studies by Monte et al.,^54^ demonstrated that PMCV-free homogenates elicited minimal non-specific inflammatory responses compared to those induced by PMCV-containing inoculum. These findings support the conclusion that PMCV is the primary driver of the observed responses. Despite our experimental limitation, the responses observed in our study are likely consistent with PMCV being the main inducer of the observed effects. Future studies should also include PMCV-negative tissue homogenate control to better isolate PMCV-specific effects and provide a more comprehensive understanding of CMS pathogenesis and host responses. Addressing all these limitations in future studies could provide a more holistic understanding of CMS pathogenesis and mitigation strategies.

## Resource availability

### Lead contact

Further information and requests for resources and reagents should be directed to and will be fulfilled by the lead contact Jaya Kumari Swain (jaya.k.swain@uit.no).

### Materials availability

This study did not generate new unique reagents.

### Data and code availability

• All data in this paper will be shared by the lead contact upon request.
• This paper does not report original code.
• Any additional information required to reanalyze the data reported in this study is available from the lead contact upon request.

## Acknowledgments

We would like to thank Øystein Evensen (Faculty of Veterinary Medicine, 10.13039/501100008119Norwegian University of Life Sciences) for providing PMCV-positive heart samples which was used as a source of PMCV for the infection experiment. We would also like to thank Tom-Ivar Eilertsen and Kenneth Bowitz Larsen (Department of Medical Biology, UiT The Arctic 10.13039/100007465University of Norway, Tromsø, Norway) for technical assistance with electron microscopy samples and Miroslava Hansen from 10.13039/501100024104Norwegian Veterinary Institute, Harstad for technical help in tissue sectioning and staining. Finally, we would like to thank Vismai Naik Thuppe and Biswajoy Ghosh from UiT for their assistance with sample processing and microscopy on experimental days and staffs at Aquaculture Research Station in Tromsø for assistance in fish maintenance. This work was supported by UiT The Arctic University of Norway thematic grant (VirtualStain project, Cristin Project ID: 2061348). 10.13039/501100005416The Research Council of Norway (grant no. 325159) is also acknowledged.

## Author contributions

Study conceptualization, A.M., K.A., R.A.D., and J.K.S.; fish handling and dissection, A.M., R.A.D., and J.K.S.; tissue digestion and cardiomyocyte purification, A.M. and J.K.S.; fluorescence microscopy, A.M., I.S.O., and D.L.W.; electron microscopy sample preparation and imaging, A.M. and R.O.; RT-qPCR, A.M.; histopathology analysis and histoscore of heart, A.S.D.; data analysis and visualization, A.M. and J.K.S.; writing – original draft, A.M. and J.K.S.; writing – reviewing and editing, A.M., R.A.D., A.S.D., and J.K.S.; funding acquisition and supervision, J.K.S. and R.A.D.

## Declaration of interests

The authors declare no competing interests.

## STAR★Methods

### Key resources table

**Table:** 

REAGENT or RESOURCE	SOURCE	IDENTIFIER
**Bacterial and virus strains**		

Piscine myocarditis virus (PMCV)	Prof. Øystein Evensen (NMBU) https://doi.org/10.1016/j.fsi.2025.110518	N/A

**Chemicals, peptides, and recombinant proteins**		

MitoTracker Green FM	ThermoFisher Scientific	Cat#M7514
CellMask Deep Red Actin Tracking Stain	ThermoFisher Scientific	Cat#A57248
Collagenase Type II	Gibco	Cat#17101015
Collagenase Type IV	Gibco	Cat#17104019
Protease XIV	Sigma-Aldrich	Cat#P5147-100MG
Leibovitz's L-15 Medium, no phenol red	ThermoFisher Scientific	Cat#21083027
SYBR Green Master Mix	Applied Biosystems	Cat#4309155
Hematoxylin solution	Sigma-Aldrich	Cat#1.05175.0500
Eosin Y-solution	Sigma-Aldrich	Cat#45380

**Experimental models: Organisms/strains**		

Atlantic salmon (Salmo salar)	Aqua Gen AS	N/A

**Oligonucleotides**		

Primer sequences	This study	See Table S1

**Software and algorithms**		

FIJI (ImageJ)	Fiji (http://fiji.sc)	RRID:SCR_002285
Graphpad PRISM v10	GraphPad (https://www.graphpad.com/features)	RRID:SCR_002798
Radius software v2.0	EMSIS GmbH (https://www.emsis.eu/products/radius)	N/A
NDP.view2	Hamamatsu Photonics (NDP.view2 Viewing software U12388-01 | Hamamatsu Photonics)	RRID:SCR_025177

**Other**		

QuantiTect Reverse Transcription Kit (200)	Qiagen	Cat#205313
RNEasy Mini Kit (250)	Qiagen	Cat#74106

### Experimental model and study participant details

#### Animal studies

Atlantic salmon (*Salmo salar* L.; standard strain provided by Aqua Gen AS) post-smolts of both sexes were sourced from the Tromsø Aquaculture Research Station (Tromsø, Norway). The fish were maintained in seawater at 12°C under continuous light (24-hour) conditions and were fed according to appetite (Nutra Olympic 3 mm, Skretting) for the duration of the study.

A total of 108 pathogen-free fish were randomly divided into a control group (*n* = 54) and an infected group (*n* = 54), with each group housed in three 500 L tanks containing 18 fish each. Before the experiment, fish were checked by routine analysis (PCR-based/histopathology). The analysis showed that the fish were free from infection by piscine myocarditis virus (PMCV), piscine orthoreovirus (PRV), infectious pancreas necrosis virus (IPNV), salmonid alpha virus (SAV), infectious anaemia virus (ISAV), salmon gill poxvirus (SGPV), avirulent infectious salmon anaemia, *Branchiomonas cysticola*, and *Candidatus clavochlamydia salmonicola* (causing epitheliocystis disease). Following a two-week acclimation period, the fish were weighed (mean ± SD: 109 ± 18 g) and then injected using an infectious salmon heart tissue homogenate (provided by Dr. Øystein Evensen, Norwegian University of Life Sciences, Ås, Norway) from a 2018 CMS outbreak (location: Farmannsøya, the diagnosis was confirmed by histopathological examination). The homogenate prepared in 0.9% saline were centrifuged and 0.45 μm filtered and confirmed by RT-qPCR to be free of SAV and PRV. Fish were anesthetized with benzocaine (40 mg/L; ACD Pharmaceuticals, Oslo, Norway) prior to injection. Infected fish received 0.1 mL of the homogenate intramuscularly on each side below the dorsal fin, while controls were injected with 0.1 mL of 0.9% saline, consistent with similar studies by Su et al. and Fritsvold et al.^55^^,^^56^ However, we acknowledge that, in addition, a tissue homogenate from PMCV-free fish, as described by Monte et al.^54^ would have been an extra control to isolate the specific effects of PMCV.

Sampling was conducted at 1, 2, 4, 6, 7, 9, 12, 14, and 16-weeks post-infection (WPI), with six fish from each group collected at each time point (Figure 1). Euthanasia was performed using benzocaine overdose (80 mg/L) followed by a blow to the head. At each sampling time point, the ventricle was excised and divided into three portions: one for RNA extraction and preserved in RNAlater for storage at –20 °C, one for histopathological scoring (only at 2, 4, 8, 12, 14 and 16 WPI), and one for enzymatic digestion to isolate single-cell cardiomyocytes. Whole ventricle tissue refers to the entire ventricular sample, including both spongiform and compact layers, while the isolated cardiomyocyte fraction refers to single-cell cardiomyocytes obtained through enzymatic digestion of the ventricular tissue.

#### Ethics statement

All experimental procedures were approved by the Norwegian Food Safety Authority (NFDA; approval ID: 28060) and conducted in compliance with the European Union Directive 2010/63/EU, as well as the current Norwegian regulations on animal welfare (FOR-1996-01-15-23).

### Method details

#### Cardiomyocyte isolation

Salmon cardiomyocytes were isolated from the ventricle tissue following a modified enzymatic digestion protocol.^7^ Briefly, the ventricle was excised aseptically, cut into small pieces and kept in perfusion buffer prior to cardiomyocyte isolation (PBS supplemented with 10 mM HEPES, 10 mM glucose, 10 mM BDM, 10 mM taurine, and 1 mM MgCl_2_). These pieces were then further minced and digested using a solution containing 0.5 mg/mL collagenase type II, 0.5 mg/mL collagenase type IV, and 0.05 mg/mL protease XIV in perfusion buffer at 15°C. Digestion was stopped once tissue was adequately digested (∼45 – 60 minutes) via addition of 5% FBS in perfusion buffer. Samples were kept on ice thereafter. Remaining cell clumps were then further broken down by drawing the solution through a syringe, filtered through a 100 μm cell strainer, and centrifuged at low speed (10 x g) to settle the myocytes at the bottom of the tube. The settled myocytes were resuspended in culture media (L15 supplemented with 5% FBS and 10 mM BDM). Cells intended for microscopy were put into microscopy dishes (Ibidi gridded μ-Dish 35 mm) and kept in a cell incubator at 15°C. The remaining cardiomyocytes were preserved in lysis buffer for RNA isolation and subsequent gene expression analysis.

#### RNA isolation, cDNA synthesis and RT-qPCR reaction

Whole ventricle tissue samples preserved in RNAlater were minced and homogenized in RLT lysis buffer with 40 mM dithiothreitol (RNEasy Mini Kit, Qiagen) using a TissueLyser II (Qiagen), and RNA was extracted using the RNEasy Mini Kit (Qiagen). Cardiomyocyte RNA was isolated using RNAqueous Micro Kit (Invitrogen), with purified cardiomyocytes stored in lysis buffer immediately after isolation. RNA concentrations were measured using a NanoDrop (Thermo Fisher Scientific) and RNA samples were stored at –80°C. cDNA was synthesized using the QuantiTect Reverse Transcription Kit (Qiagen) and stored at –20 °C. RT-qPCR was performed using SYBR Green Master Mix (Applied Biosystems) in 15 μL reaction volume containing 6 μL of 1:25 diluted cDNA, in duplicate, on a 7500 Fast Real-Time PCR System (Applied Biosystems). The cycling conditions consisted of an initial denaturation step at 95 °C for10 min, followed by 40 cycles of 15 s at 95 °C and 60 s at 60 °C, concluding with a melt curve analysis. The *18s* gene was used as an endogenous control for normalization. Gene expression fold-changes were calculated using ΔΔCt method according to Pfaffl,^57^ while viral RNA level was quantified by targeting the PMCV ORF2 region, with up-regulation measured relative to the limit of PMCV RNA detection (ct value of 36, where specific ORF2 amplification first observed) and further normalized with18S. The Ct threshold was set to 0.1 for all analyzed genes. Primers used are listed in Table S1. Due to the small size of the fish and heart, the isolation of RNA from isolated cardiomyocytes yielded insufficient RNA for RT-qPCR analysis throughout the time point and especially at 1 and 4 WPI.

#### Fluorescence microscopy (FM) of live cardiomyocytes

Cells were stained with MitoTracker Green FM (Thermo Fisher Scientific) at a 1:10,000 dilution and CellMask Deep Red Actin (Thermo Fisher Scientific) at a 1:2,000 dilution, both prepared in perfusion buffer according to manufacturer’s recommendations. Staining was performed for 30 minutes at 15°C. Following staining, isolated cells were washed three times with PBS, placed under phenol red-free L-15 culture media and imaged using a DeltaVision Elite deconvolution microscope at 60x with oil immersion (NA = 1.42). An image stack was taken, with steps of 250 nm between images. Imaging was performed with an exposure time of 30 ms for the MitoTracker channel 10 ms for the actin channels. Deconvolution of the acquired image stacks was performed using DeltaVision software prior to further analysis.

#### Transmission electron microscopy (TEM)

Isolated cells were cultured for 18 hours on gridded #1.5 polymer coverslips in 35 mm dishes (Ibidi gridded μ-Dish 35 mm) and fixed on ice with 4% formaldehyde and 1% glutaraldehyde in marine PHEM buffer containing 9% sucrose. For TEM preparation, samples were sequentially treated with 0.05% malachite green (Sigma-Aldrich), 1% osmium tetroxide (Electron Microscopy Sciences) with 0.8% potassium ferricyanide (Sigma-Aldrich), 1% tannic acid (Electron Microscopy Sciences), and 1% uranyl acetate (Electron Microscopy Sciences). This was followed by graded ethanol dehydration and embedding in epoxy resin (Agar Scientific) using a Pelco BioWave microwave processor (Ted Pella, Inc.). Resin blocks were polymerized at 60 °C for 48 h. After polymerization, blocks were trimmed using a glass knife on a UC6 ultramicrotome (Leica Microsystems) and sectioned into 70 nm ultrathin slices with a 35° ultra-knife (Diatome). Sections were collected on slot grids and imaged at 15,000x magnification on a Hitachi HT7800 TEM at 100 kV using a Xarosa CMOS camera, with scale bars and contrast adjustment embedded using the Radius software v2.0 (EMSIS). Protocol adapted from Godtliebsen et al.^58^

#### Image analysis

We analysed FM and TEM images to investigate mitochondrial morphology in isolated ventricular cardiomyocytes from both the uninfected and PMCV infected fish.

Fluorescence images were manually segmented into single cell images prior to further processing. To minimize out-of-plane signal, a variance filter was used to mask signal in the MitoTracker channel, converting low-variance regions into NaN pixels (Figure 2A). In parallel, a two-dimensional Difference-of-Gaussians filter was applied to enhance image contrast, with NaN pixels excluded from the contrast-enhanced images. Mitochondria were segmented using the Otsu algorithm and analyzed with the built-in “Analyze Particles” feature in ImageJ. To avoid repeated measurements of the same mitochondria, only every third slice was analyzed for each cell. Total number of mitochondria per cell was calculated. To measure the morphometrics of mitochondrial sub-types, fluorescent images were further segmented based on spatial localization. Perinuclear (PN) mitochondria were analyzed by segmenting regions within 2 μm of the nucleus. For intermyofibrillar (IMF) mitochondria, segmentation excluded the PN region and the cell termini (defined as 5-15 micrometers from each tip of the cell) while maximizing the number of mitochondria analyzed (Figure 2B). Percentage of PN mitochondria was calculated from the segmented part of the nucleus region compared to the total number of mitochondria per cell. All image processing and analysis were performed using imageJ. In total, 234 and 233 cardiomyocytes were analyzed in the infected and uninfected groups, respectively, originating from 1-3 fish per timepoint.

Mitochondria in TEM images were segmented manually (Figure 2C) and classified according to their spatial location within the cardiomyocyte (as shown in Figure 2B) into either a perinuclear (PM) or intermyofibrillar (IMF) mitochondrial groups depending on distance from the nucleus. Segmented mitochondria were further analyzed using the “Analyze Particles” feature in ImageJ. In the control group, a total of 3129 mitochondria were analyzed from 11 cardiomyocytes across 161 regions of interest (ROIs). In the infected group, 1979 mitochondria were analyzed from 11 cells, across 100 ROIs.

Mitochondrial morphometrics for both FM and TEM images were quantified using the shape descriptors of individual mitochondria measured in 2D - area, aspect ratio (*AR*), roundness (*R)*, circularity and Feret’s minimum diameter (*FMD*) as shown in Figure 2D. The mitochondrial area (μm^2^) was used as a measure of overall size. Aspect ratio quantified mitochondrial elongation, reflecting the balance between fission and fusion events. Roundness and circularity was included as a complementary metric to assess deviations from an elongated tubular morphology and smoothness, with higher values indicating more spherical or fragmented mitochondria, typically associated with increased fission or swelling.^43^^,^^59^ Feret’s minimum diameter was used as a proxy for mitochondrial thickness and short-axis swelling, which is sensitive to matrix expansion and early permeability transition like changes.

#### Histopathological evaluation of CMS

Heart samples (*n*=6) from uninfected and PMCV infected group were collected at 2, 4, 6, 8, 12, 14 and 16 days post challenge for histopathological analysis. The samples were fixed in 10% buffered formalin for a minimum of 48 hours to ensure proper fixation. The specimens were subsequently embedded in paraffin wax, sectioned, and stained with haematoxylin and eosin (H&E) following standard histological procedures.^60^ The stained slides were scanned at 40X using Hamamatsu NanoZoomer S360 digital slide scanner and analysed with NDP.view2 software (Hamamatsu Photonics K.K., Hamamatsu City, Japan). Histopathological changes in heart sections were scored as described in Kavaliauskiene et al. 2022.^61^ In brief, a general scoring scheme was assigned, scoring distribution of inflammation, degeneration and necrosis of cardiomyocytes and associated tissue in the atrium, spongiosum of the ventricle, compactum of the ventricle and epicardium, respectively. The distribution of lesions in each compartment were scored as follows: score 0 = no lesions, score 0.5 = <12.5 % of the compartment affected, score 1 = 12,5–25 % of the compartment affected, score 2 = 25–50 % of the compartment affected, score 3 = 50–75 % of the compartment affected and score 4 = >75 % of the compartment affected. Scoring was performed blinded, meaning that the identity of the slide (control and infected) was unknown to the histopathologist.

### Quantification and statistical analysis

Gene expression data were analyzed based on their distribution, which was assessed using the Shapiro-Wilk normality test. Parametric t-tests were applied to normal or log-normally distributed data, while the Mann-Whitney U test was used for non-normal data, histopathology score data and microscopy results. Correlation analysis between individual gene expression and viral RNA expression (log2 fold-change relative to 18S) was performed using linear regression on data pooled across all time points. Spearman’s rank correlation was used to evaluate correlations across all gene expression data. Statistical analyses and graphing were performed in GraphPad Prism v10. For all statistical analyses: ns = not significant, ∗*p* < 0.05, ∗∗*p* < 0.005, ∗∗∗*p* < 0.0005, and ∗∗∗∗*p* < 0.00005. The data are expressed as mean ± standard error of the mean (SEM) or as median with whiskers (extending to the minimum and maximum values), as indicated. The number of experimental replicates for each assay is specified in the corresponding figure legends.
