# Supplementary material for: Mitochondrial dynamics and antiviral responses in Atlantic salmon cardiomyocytes during piscine myocarditis virus infection
Source: iScience. 2026 Jul 18;29(8):116831. doi: 10.1016/j.isci.2026.116831 (PMC13393163; doi:10.1016/j.isci.2026.116831)
Supplement: Document S1. Figures S1–S4 and Table S1 [file mmc1.pdf]

iScience, Volume *29*

Supplemental information

Mitochondrial dynamics and antiviral responses in Atlantic salmon cardiomyocytes during piscine myocarditis virus infection

Antoni Malachowski, Deanna Lynn Wolfson, Randi Olsen, Alf Seljenes Dalum, Ida Sundvor Opstad, Krishna Agarwal, Roy Ambli Dalmo, and Jaya Kumari Swain

Supplemental Figures

Supplemental Figure 1

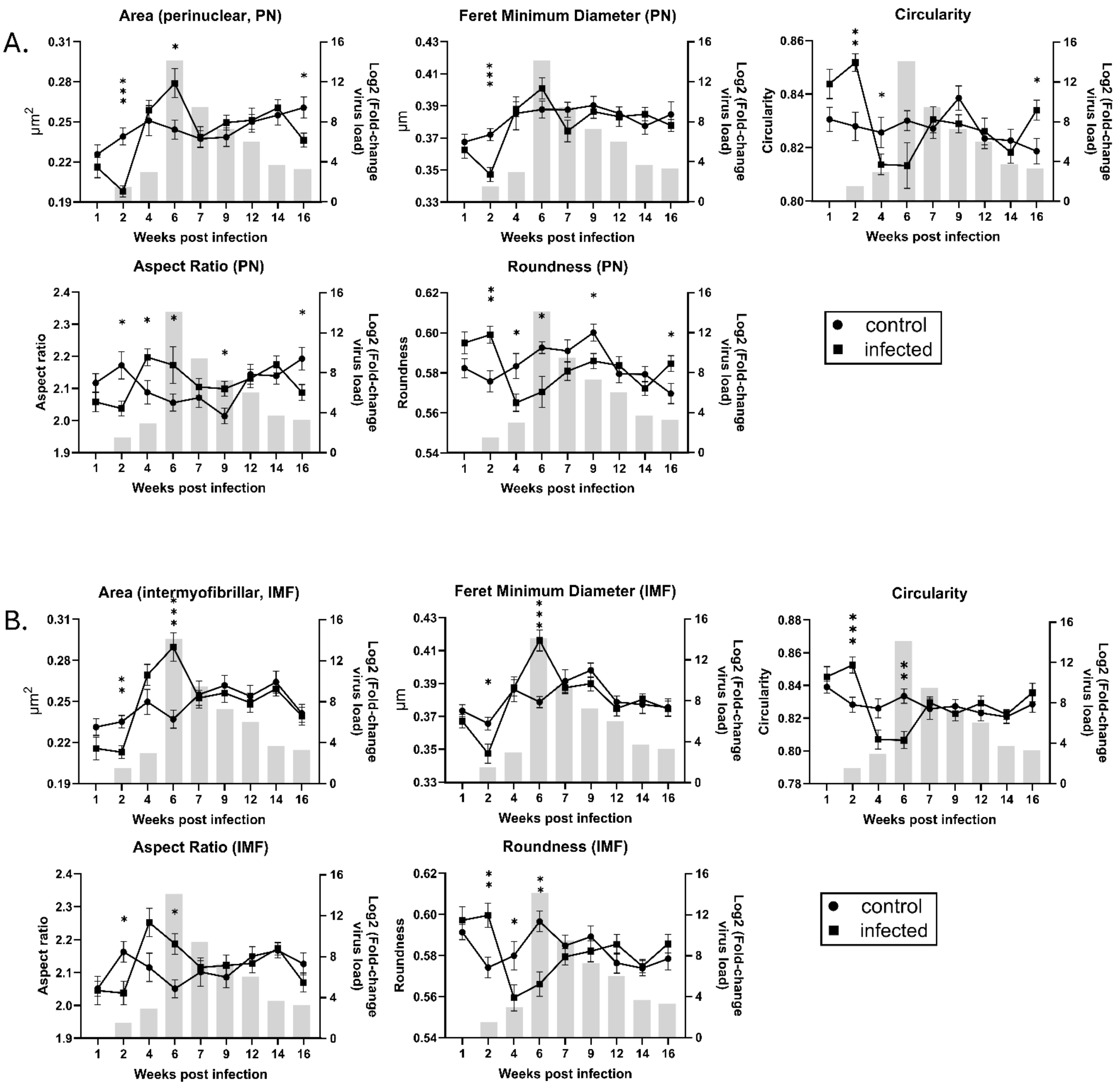

Figure S1. Quantitative analysis of mitochondrial subpopulations in ventricular cardiomyocytes, related to Figure 7. Cardiomyocytes were isolated from ventricle tissue, purified, and cultured at multiple time points following PMCV infection, then stained with MitoTracker Green for imaging. (A) Quantitative analysis of perinuclear (PN) mitochondria in isolated cardiomyocytes. A total of 201 cardiomyocytes were analyzed in the infected group and 214 in the uninfected group, with 1–3 fish sampled per group per time point. (B) Quantitative analysis of intermyofibrillar (IMF) mitochondria in isolated cardiomyocytes. A total of 209 cardiomyocytes were analyzed in the infected group and 215 in the uninfected group, with 1–3 fish sampled per group per time point.

For both panels, mitochondrial sizes were calculated per cell, with mean ± SEM values shown as points on the line graph (left y-axis). Virus RNA levels, measured via RT-qPCR, are displayed as bars in the background (right y-axis). Statistical significance for both panels is indicated as $*p < 0.05$, $**p < 0.005$, and $***p < 0.0005$, calculated using the Mann-Whitney U test.

Supplemental Figure 2.

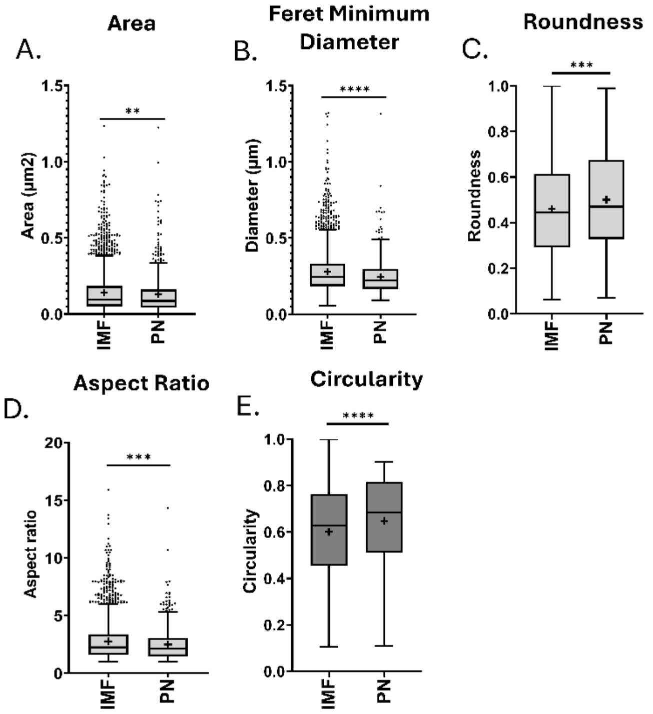

Figure S2. Comparison of intermyofibrillar and perinuclear mitochondria in uninfected cardiomyocytes using electron microscopy related to Figure 8. Atlantic salmon cardiomyocytes were isolated from the ventricle tissue, purified, cultured, fixed, and embedded for electron microscopy. Mitochondria were classified based on their distance from the nucleus: those within 2 µm were categorized as perinuclear (PN), while those beyond this threshold were classified as intermyofibrillar (IMF). Each data point represents an individual mitochondrion. Statistical significance is indicated as **$p < 0.005$, ***$p < 0.0005$, and ****$p < 0.00005$, calculated using the Mann-Whitney U test. Box plots display the median, interquartile range (IQR), whiskers extending to data within 1.5×IQR, and individual outliers shown as points. The group means is indicated by a plus symbol (+).

Supplemental Figure 3.

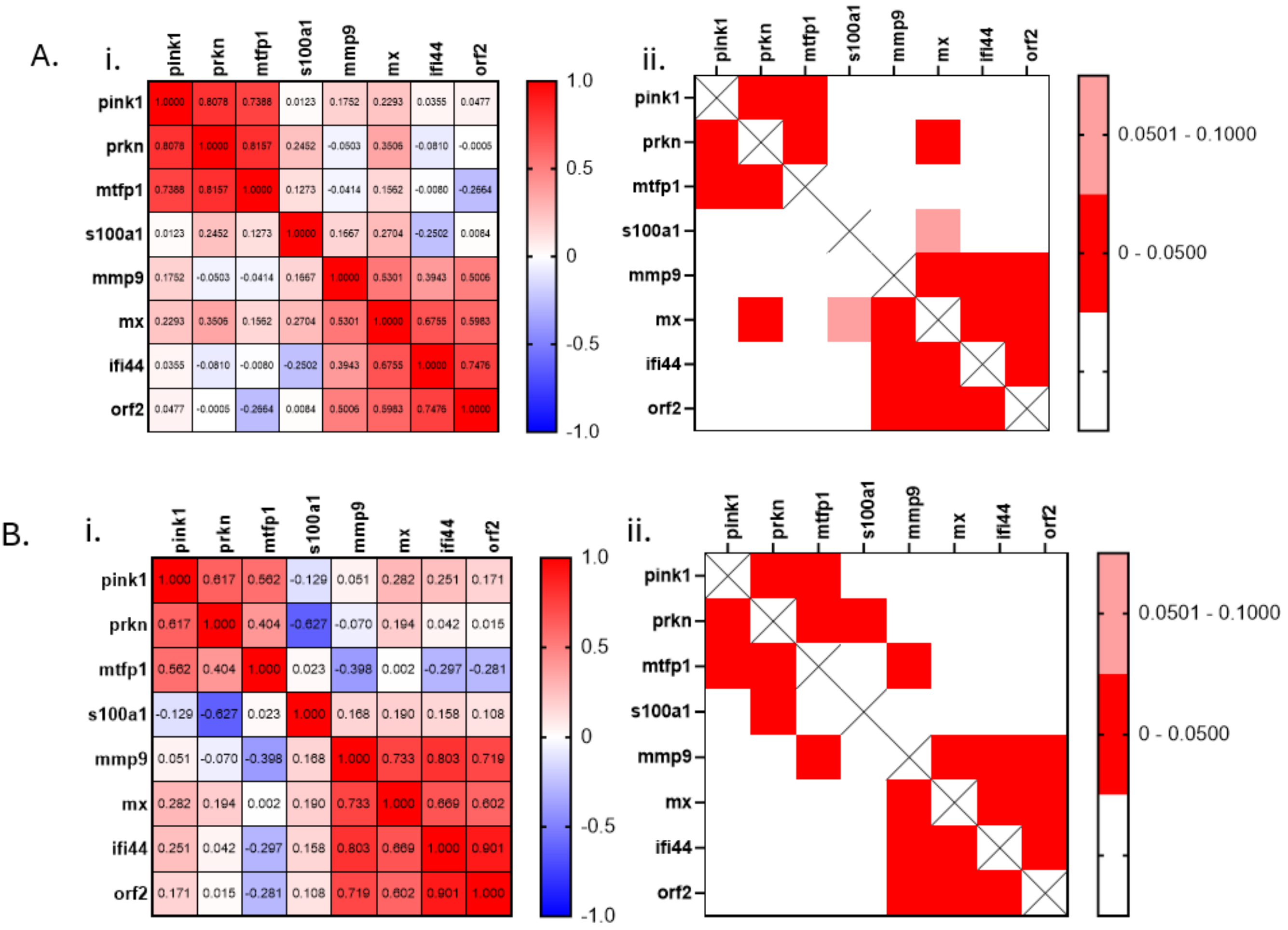

Figure S3. Correlation of gene expression in salmon heart samples, related to Figures 9-12. Gene expression data obtained by RT-qPCR across all time points were pooled and subjected to Spearman correlation analysis using GraphPad Prism (v10). (A) Whole ventricle tissue samples. (B) Isolated cardiomyocyte samples. For each panel, (i) Spearman correlation heatmaps display the correlation coefficients between gene pairs as numerical values within each cell, and (ii) statistical significance was calculated in GraphPad Prism and categorized into two levels (p > 0.05 and p > 0.1).

Supplemental Figure 4.

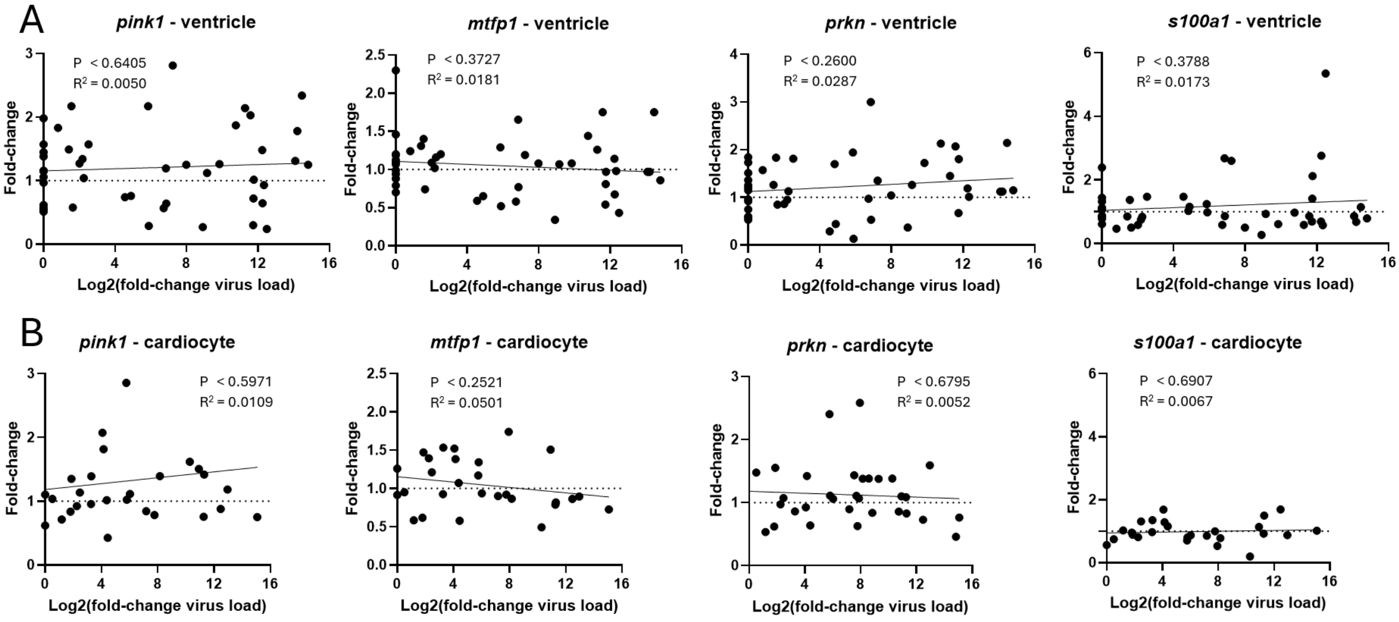

Figure S4. Correlation of mitochondrial gene markers with virus RNA expression levels in whole ventricle tissue and isolated cardiomyocyte samples, related to Figures 10 and 12. Linear regression analysis was performed to evaluate the correlation between gene marker expression and viral RNA expression (log2 fold-change) using data pooled across all time points. (A) Results from whole ventricle tissue samples (B) Results from isolated cardiomyocytes. The statistical significance of the regression model and its deviation from the null hypothesis were assessed using linear regression analysis. Relevant p-values and R^2 values are provided in the inset.

Supplemental Tables

Supplemental Table 1.

Gene Target	Name	Fwd (5-3')	Rev (5-3')	Efficiency
18S ribosomal RNA	*18s*	TGTGCCGCTAGAGGTGAAATT	CGAACCTCCGACTTTCGTTCT	87.131
Interferon induced protein 44	*ifi44*	AGTGATCCTGACGTGGGCCAG	GGGCACCACTTGTTGTCCGATT	82.928
Matrix metalloprotease 9	*mmp9*	TGGAGAGAACTACTGGAGGCTGGA	CCGACAGAAGTAGATGTGGCCCTT	102.831
Mitochondrial fission process 1	*mtfp1*	AGCATGGTGACAACCCAGGGA	GGATAGCGACCGAGGCCAGG	92.253
Myxovirus-resistant gene (Mx)	*mx*	TGCAACCACAGAGGCTTTGAA	GGCTTGGTCAGGATGCCTAAT	102.441
PMCV open reading frame 2	*orf2*	GGAAGCAGAAGTGGTGGAGCGT	CCGGTTTTGCGCCCTTCGTC	96.846
PTEN-induced kinase 1	*pink1*	GCGGCTCGCCAAGAAACAGA	CCTGGCATGTCGCAGCACTC	97.64
Parkin RBR E3 Ubiquitin Protein Ligase	*prkn*	GGCACCCTCCAGGGCTATGA	GAGGCTGTCCACCCCTCGAC	79.908
S100 Calcium Binding Protein A1	*s100a1*	CCAGAAGGACCCAGCTGCCA	GGAGAGGCCCACCACCAGAG	100.962

Table S1. Primer sequence and efficiency, related to Figures 9-12. Primers were ordered from Sigma Aldrich. To determine efficiency, Ct values were plotted against the template concentration, and a linear regression line was fitted using 7500 software.
